# Medicine and Charity in Eighteenth-century Northumberland: The Early Years of the Bamburgh Castle Dispensary and Surgery, *c*. 1772–1802

**DOI:** 10.1093/shm/hkw008

**Published:** 2016-05-02

**Authors:** Alun Withey

**Keywords:** medicine, charity, dispensaries, hospitals, enlightenment

## Abstract

In 1772 in Bamburgh Castle, Northumberland, a charitable institution was established by Dr John Sharp to offer medical provision to the poor of the parish, which was remote from the Newcastle and Edinburgh Infirmaries. Unlike urban institutions, which have dominated hospital historiography, the Bamburgh dispensary was small, occupying only a few rooms in the castle, and situated in a remote, coastal location. And yet, at its height, the Bamburgh dispensary treated thousands of patients per year, often exceeding dispensaries in large towns, and was equipped with the latest medical technologies. Unlike the majority of infirmaries and dispensaries it was not funded by subscription, nor run by governors, but was entirely funded by the Lord Crewe Trust, and administered by Dr Sharp. While Bamburgh is certainly an anomaly, it raises new questions about voluntary institutional medical provision for rural populations, and forms of medical philanthropy.

In the summer of 1772, Dr John Sharp, archdeacon of Northumberland and renowned philanthropist, set out plans for a new medical institution. Its stated aims were to ‘give advice, administer medicines, dress the sores of all such poor persons as shall apply for such assistance … and do everything that shall be done in a regular infirmary’.^1^ It was free to anyone unable to pay for their treatment elsewhere, ‘whether English, Scotch or Foreigners’.^2^ Capable of treating thousands every year, it boasted high quality medical equipment and provisions, mechanical bathing facilities, an infirmary carriage and large store of drugs. In all respects this was a facility worthy of any large, aspirational Georgian town. Its aim to provide for the ‘sick and lame objects’ of the community was a veritable mission statement for eighteenth-century civic charitable worthiness. But this institution was not located in any town; it was situated in Bamburgh Castle, a formerly dilapidated medieval leviathan on the rugged Northumberland coast. Unlike virtually every other hospital or dispensary in the country there were no subscribers or board of governors. Instead the facility was funded entirely through monies from a general charitable trust established by the late Nathaniel Lord Crewe, under the management of one man. In the last quarter of the eighteenth century, Bamburgh Castle treated as many patients as some of the largest dispensaries in provincial England. It was well equipped and arguably more accessible than most, allowing entry to any whose status as a ‘worthy object’ could be attested to through a signed certificate from local clergy. In all respects it was an extraordinary experiment, one that saw a metropolitan model of medical provision transposed onto the unlikely setting of a remote medieval castle.

Much historiographical attention has focused upon the polymorphous social, economic and political, as well as medical, meanings of eighteenth-century institutions.^3^ Hospitals have been firmly located within the nascent civic, urbane and polite milieu of Georgian towns, and changing models of philanthropy. In many respects hospitals were the ultimate expression of practical benevolence. They were highly prominent and visible edifices within urban landscapes, while also providing a hub of medical provision both within the town and its hinterlands. In providing treatment gratis for large numbers of the poor, hospitals served a practical need in supplementing the medical provision of the poor law. Voluntary hospitals tended to cater for those above the level of requiring poor relief (or reluctant to call upon it) while lacking resources to fund their own medical care. But what was institutional health care like in rural areas? Away from the usual models of subscriber funding, and nexus of urban philanthropy and municipal charity, how were rural institutions funded? Equally, away from densely populated urban centres, who used them?

## Eighteenth-Century Medical Institutions

The primary means of funding eighteenth-century hospitals was through subscription. Subscribers gained the right to recommend patients, and also to obtain administrative privileges such as a governorship, whilst various benefits of benefaction were used as incentives to entice donors.^4^ Equally, donors gained the moral satisfaction of giving coupled with the practical benefits of involvement in a prominent urban administrative entity. Many were well equipped, staffed with professional practitioners and offered treatments usually beyond the reach of plebeian patients. In addition, however, they have been seen as performing a socially cohesive role, reinforcing the social order by bringing together both the upper and lower orders, and healing rifts within elite ranks.^5^ Or, as Roy Porter famously put it, hospitals ‘paper[ed] over the cracks between patricians and plebeians’.^6^

The requirement for a letter of recommendation served to regulate numbers. It also encouraged the gratitude, and obeisance, of poor patients. Whilst the presence of an infirmary enhanced the fabric of the town, it also reinforced civic identity and stressed the preeminent role of the town within the broader county framework.^7^ Others, like Patrick Wallis, stress the political elements underlying hospital foundation, arguing that hospital establishment could be a site of factional conflict.^8^ In all cases, however, the overwhelming focus is upon the urban nature of eighteenth-century hospital provision and its place within the nexus of civic virtue.

Alongside hospitals, dispensaries were the other main form of institutional medical provision. While the main spate of hospital construction occurred in the first half of the eighteenth century, the dispensary movement was a feature of its last quarter. Dispensaries were benevolent institutions founded to offer shelter and medical attention to the sick poor.^9^ Between 1769 and 1792, 21 were opened in London alone.^10^ In the last quarter of the eighteenth century, a further 22 dispensaries were established around Britain, the majority of which were located in the north of England.^11^ Whilst some dispensaries had a few beds, their services were mainly for out-patients. These included routine treatments and procedures, dispensing medicines, midwifery services to the poor and sometimes other services such as bathing facilities.^12^ According to Robert Kilpatrick, dispensaries were usually founded by medical practitioners, and often by dissenting physicians, whose moral and religious beliefs shaped their beliefs regarding fair and charitable treatment for the poor.^13^ Like voluntary hospitals, however, dispensaries were characteristically urban institutions, generally sited in large towns, and sometimes, as in Newcastle, in places where there was already an infirmary.^14^ Also like voluntary hospitals, dispensaries relied upon subscriptions, donations and legacies and were generally administered on similar lines. Dispensary historiography has generally focused on London, with provincial dispensaries receiving less attention.^15^

However, the records of Bamburgh offer a unique opportunity to broaden and deepen our knowledge of eighteenth-century institutional medical provision. The development of medical institutions has tended to be portrayed in linear terms. Hospitals and infirmaries emerged first in London, before moving out into the provinces. A dispensary movement followed after 1770, again beginning in London and mapping across English towns. Bamburgh, founded in 1772, and almost completely overlooked in hospital historiography, could lay solid claim to be the first of the provincial dispensaries and yet fits neither the usual location nor model. It reminds us of the dangers of linear narratives and of selectivity, in requiring institutions to fit neat labels. In fact its very form and location questions the validity of demarcation lines between different types of institution. The focus in hospital historiography, for example, has been upon in-patients, with dispensaries assumed to have been the providers of out-patient care. In reality, however, there was an overlap. Hospitals also treated many out-patients and some housed dispensaries. In urban areas there was sufficient demand for dispensaries to provide out-patient care above and beyond the town hospital. In Bamburgh, serving a disconnected rural community, there was no need to differentiate; the institution could be both infirmary and dispensary and was referred to as both by contemporaries.

The nomenclature of medical institutions is also rarely considered. The term ‘infirmary’ for example, originally referred simply to a room where sick people could be housed. ‘Hospital’ likewise shifted in meaning across time from its original Latin etymology of a charitable building or apartment for guests. Originally spatial terms, they have come to be used, often uncritically, to describe and define institutional function and provision. The various uses of medical space at Bamburgh, and variable terminology used, highlight the diversity of medical activities that could be housed under a single roof, defying neat compartmentalisation.

Perhaps most importantly, the establishment and management of the medical institution at Bamburgh broadens our understanding of accepted forms of medical philanthropy, and especially funding mechanisms. The historiography of British hospitals and dispensaries has focused upon subscriber-led models. The trustees of Bamburgh, and the Archdeacon John Sharp in particular, however, were able to act with relative autonomy, without the need for public funds or governing bodies. As Bamburgh suggests, formal hospital and dispensary services were not always necessarily connected with civic ambitions. Health provision could be divested from its usual context of urban and civic development. In shifting hospital provision away from the urban context this article sheds new light on the institutional care in rural areas, along with the establishment, administration and function of medical philanthropy.

## The Making of a Medical Charity

Key to understanding the unique nature of Bamburgh is its patron, Dr John Sharp. Sharp was part of a large and ecclesiastical family. His father was Thomas Sharp, the archdeacon of Northumberland, and his grandfather the Archbishop of York. His brother Granville was a prominent anti-slavery campaigner, while another brother, William, was surgeon to St Bartholomew’s hospital in London. John Sharp was a Cambridge graduate and held various church offices before, in 1762, becoming archdeacon of Northumberland. In 1773 he became the perpetual curate of Bamburgh, taking up residence in the castle. Sharp’s beliefs in charity were shaped by his religious views. According to Françoise Deconinck-Brossard, Sharp held latitudinarian views, combining practical religion with a strong moral focus.^16^ Such views encouraged his beliefs in practical benevolence, and partly explain his willingness to be physically, as well as financially, involved in efforts to improve the lot of the poor. He was also an astute businessman. During his tenure as manager of the Tweed Fisheries in the 1750s, their income rose fivefold and the value of the business increased from £150 to £492.^17^

Although Sharp was relatively wealthy, it was his role within the Lord Crewe charity that provided the financial heft to his Bamburgh project. In 1721, Nathaniel Crewe, the third Baron Crewe and Bishop of Durham, had died leaving a vast fortune based on property. All Crewe’s properties in the north east of England, including Bamburgh Castle, were left in trust. Other bequests were made to Oxford Colleges and various other provisions made for the poor and needy. Crewe’s will stipulated that any surplus revenue should be given over to charitable use at the discretion of the trustees, a surplus that by 1731 had grown to over £1,300. In 1758, John Sharp was appointed trustee upon the death of his father. Sharp undertook various repairs to the castle towers and also made preparations to give over part of the building for charitable use, notably a school for poor children and a corn charity. Sharp was in fact central to the establishment of the hospital as an expression of his own charitable beliefs. Contemporaries remarked upon his munificence. In 1776 William Hutchinson, author of a study of Northumberland, commented that Sharp ‘has his eye upon every new channel by which he may give relief or consolation to his suffering fellow-creatures’.^18^ A contemporary portrait depicts Sharp, apparently inside the castle, with one hand upon what appear to be plans for building work in the castle, the other open as two supplicating paupers approach him.^19^

According to a nineteenth-century account, Sharp’s own explanation for the foundation of his ‘little hospital’ was the death of the master of a vessel wrecked on the treacherous rocks next to the castle, who died not in the sea but due to the ‘damp bed in the village’ where he was brought.^20^ In August 1772 the rationale for a new hospital was laid out.

‘Whereas the poor in the neighbourhood of Bamburgh being at the distance of near 50 miles from the Newcastle Infirmary, and at a still greater distance from the Infirmary at Edinburgh,are many of them so very necessitous, that they can neither pay a surgeon for attendance or advice, or be at the necessary expence (sic) of Drugs when sick’.^21^

‘Mission statements’ like this were common in the establishment of voluntary hospitals and used a variety of tropes to emphasise the lack or inadequacy of existing medical provision. In 1719 in Westminster, Henry Hoare noted the ‘great numbers of sick persons in this city [who] languish for want of necessaries’.^22^ In Edinburgh in 1730 the lack of available post-treatment care for the sick poor, and the opportunity to provide medical training to practitioners, were used as leverage for the establishment of an infirmary.^23^ The ‘provision of an edifice … for the reception of poor patients’, and the simple lack of existing facilities within the town and county of Hereford, were among the reasons cited by Thomas Talbot in his entreaty of 1774.^24^

The establishment of Bamburgh fits well with the shifting ‘climate of ideas’ surrounding charitable provision in the 1770s. In the second half of the eighteenth century, philosophers and philanthropists alike began to question the most suitable forms of institutional provision for the poor, and especially that of long-term, residential care. Whilst the period between 1750 and 1770 had seen the rise of lying-in hospitals such as the Lock, the Magdalen and the Foundling Hospital, concerns were beginning to be raised about the encouragement of dependency and the potential for the abuse of the system.^25^ As a result, some institutions saw dramatic falls in their numbers of subscribers. By contrast, medical institutions that offered out-patient relief, such as dispensaries, were entering something of a golden age. Unlike hospitals and infirmaries, which were often large and imposing structures, dispensaries were small, often occupying existing buildings, and had few beds. Moreover, outside London, they were almost overwhelmingly a feature of northern England.^26^ The establishment of Bamburgh, a small institution predominantly for out-patients, fits perfectly within the timescale of these changes.

Sharp’s involvement also supports Donna Andrew’s arguments regarding the increasing involvement of the clergy in philanthropist arguments about the nature of charity in the 1770s. Prominent clergy such as Josiah Tucker and William Paley, the dean of Gloucester, were actively encouraging a greater congruity between religious and economic theories of society.^27^ Sharp’s project was consistent with ideas about the extent to which charitable donors and patrons should give of their own time and attention, as well as their money.^28^

Perhaps the first question to address is what exactly Sharp’s new institution was, in order to understand where Bamburgh sits in the history of medical charity. It seems clear from the outset that the institution at Bamburgh defies neat categorisation, and this raises interesting questions both about the use of medical space, and the terminologies used to describe it. As Jonathan Reinarz and Leonard Schwarz point out in their discussion of workhouses, historians have often been too uncritical in their use of terms to describe institutions.^29^ The term ‘hospital’, for example, is often interchangeable with ‘infirmary’ to describe the large-scale, multi-bedded inpatient institutions, with medical staff, often training facilities and provision for surgical procedures. Likewise, discussions of dispensaries have emphasised their distinctiveness as smaller, out-patient institutions, with few beds and a small number of staff. This is not to say that the clear differences between hospitals and dispensaries, in terms of form and function, should be elided or removed. It is perfectly clear that contemporaries used terms both to identify and to distinguish between different types of medical institution, such as the ‘London Hospital’, the ‘York Dispensary’ and so on. It is also worth noting, however, that there could be more overlap than has previously been acknowledged. Contemporary definitions of institutional terms often emphasised the use of space and form, as well as broader function. The term ‘hospital’, for example, as defined in Ephraim Chambers’ 1778 dictionary, emphasised both form and function. It was ‘a place or building erected, endowed or supported by charitable contributions for the poor, aged, infirm [and] sick …’.^30^ Further entries were dedicated to individual hospitals, often praising their size and architecture; Greenwich, for example, was lauded for its ‘magnificence and spaciousness’.^31^ By contrast, Chambers’ definition for ‘Infirmary’ was far shorter and less defined. It was simply ‘a place where the sick and weak belonging to any society are disposed, either for nursing or cure’.^32^ As such it could be a space *within* a hospital. The entry for the Royal Hospital in London made this explicit, stating that the infirmary occupied ‘a wing or outbuilding’ of the hospital.^33^ So was this a hospital, an infirmary or both? To use the terms interchangeably (and unquestioningly) is perhaps to misunderstand the spatial demarcation of different medical functions within one building. Interestingly, too, given that many had already been established by the time of its publication, Chambers’ dictionary had no individual entry for ‘dispensary’. The 1797 *Encyclopaedia Britannica*, however, defined it as ‘a magazine or office for selling medicines at prime cost to the poor’, stressing functionality rather than spatiality.^34^ Again, dispensaries could form a physical part of hospitals rather than necessarily being separate entities. Irvine Loudon in fact identified 19 examples of dispensaries and hospitals being combined.^35^

The use of space at Bamburgh demonstrates a multi-purpose medical institution, shaped both by the physical fabric of the building and the financial freedom afforded by the charity. Free to distribute funds as they saw fit, and with the huge space of the castle at their disposal, Sharp and the Bamburgh trustees neither had to create a bespoke building, nor restrict it to a single purpose. Contemporaries indeed referred to it using various terms. As originally conceived, the most common term was ‘infirmary’. It was, as noted above, intended to perform all the functions of a ‘*regular* infirmary’. In one source Sharp even referred to it as ‘my little hospital’ whereas an early nineteenth-century gazetteer referred to it as ‘a room fitted up with medicines as an infirmary’.^36^

However, other uses were noted too. In one part of the building was ‘the surgery’, located ‘in one of the lower rooms of the castle’.^37^ Another room was effectively used as an apothecary store with drugs ‘made in some proper place to contain them’.^38^ Elsewhere the multiform medical functions at the castle were more explicit. In an (unfortunately undated) manuscript by Sharp, listing the charitable activities at Bamburgh, there were three separate entries. ‘A General Dispensary for the Poor … A General Surgery for the Poor … [and] An Infirmary for particular objects viz, for sick & wounded sailors who come ashore on that coast, also for such persons as have occasion to use the Hot Bath, & for some particular cases and now & then for accidents’.^39^ A surviving headstone at the castle has the single word ‘Dispensary’, suggesting that it was located over the doorway to a particular room, or section, of the castle, rather than encapsulating the whole. Such definitional looseness reflects the fact that Bamburgh simply does not fit the usual framework of a charity hospital. Previous studies of hospitals have laid emphasis upon the imposing, purpose-built, single purpose, urban edifices that usually marked out these institutions. Bamburgh was instead a small, multi-purpose medical entity occupying different spaces within a broader charitable institution.

In many respects, Bamburgh most resembled the common definition of a dispensary. It mainly treated out-patients, had few beds, had a resident apothecary and was not purpose built. It undertook practical treatment of a range of different conditions, from fevers to cuts and bruises, it was more inclusive than many hospitals in the types of conditions it treated and, in line with other dispensaries, also ran a programme of inoculation against smallpox. It also fits the model of some London dispensaries founded and run privately by individuals, such as George Armstrong’s Dispensary for the Infant Poor, and John Bunnell Davies’ Universal Dispensary for Children, both of which utilised existing buildings.^40^ And yet it deviated from the usual dispensary model. While some dispensaries did utilise existing spaces, this was generally an expedient while permanent premises were built or located. Even contemporary observers were perplexed by the multiform nature of the charitable activities at Bamburgh, and the lack of subscribers. It was, in effect, a self-contained welfare state in miniature. As William Hutchinson commented at the time, ‘So extensive a charity from a private bounty is singular’.^41^

## Staffing and Governance

If the use of space at Bamburgh differed from the usual format, its means of governance and staffing was equally distinct. At the time of its establishment there were four trustees; Dr John Sharp; Robert Lowth, the Bishop of Oxford; the reverend John Rotherham, of Houghton Le Spring; and a Dr Douglas, rector of Stainton, Durham. Without a governing body, daily administration of Sharp’s entire Bamburgh charity, including the infirmary, was the responsibility of George Hall of Hartburn, a castle resident and trusted employee of Sharp. In May 1775, Hall was appointed as the salaried bailiff of the manor of Bamburgh, joining two other deputy bailiffs and a constable.^42^ A surgeon was appointed in August 1772 and paid half a guinea to attend for three hours on Saturday mornings to ‘give advice, administer medicines and dress the sores of all such poor persons as shall apply for such assistance’ and to perform all other necessary operations and procedures. …’^43^ He was assisted by a Mrs Rafton who lived in the castle, and was charged with looking after drugs, keeping the books and general duties. A second assistant, John Robson, was paid eighteen pence per week for assisting the surgeon, preparing medicines and ‘doing the more laborious work’.^44^ Both were expected to be sufficiently knowledgeable to act in the surgeon’s absence or support him during busy times.

One of the original castle surgeons was a Dr Turton—possibly Thomas Turton, originally of Harfield in Yorkshire, although little is recorded of him in the accounts.^45^ When Turton resigned in 1774 the posts were most probably advertised, although no records of such an advertisement can be found in either the Newcastle or London newspapers. Nevertheless, letters to Sharp from practitioners offering their services arrived from across the country, suggesting that the institution was in fact already well known and regarded in medical networks of north east England. In some cases, it was clear that word of mouth had played a part. In June 1774, Arthur Gairs, a surgeon from Alnwick in Northumberland, was ‘informed that the place of Surgeon-Apothecary for the charity of Bambro’ castle is now vacant’, offering him to visit Sharp to discuss his suitability.^46^ Others used intermediaries to attest to their good character and skill. Reverend Potter from Wallend talked up James Bower as a candidate for the post, ‘a sober, industrious and well-principled young man … who wants nothing but more experience to complete him’.^47^ But it also appears that word had spread far beyond the Northumbrian borders. Letters from William Sharp also suggest that potential candidates were approaching him in London about the position, suggesting that he may have been active in promoting it there.^48^ The position was ultimately filled by William Cockayne, likely the same surgeon originally of Mile End, Middlesex, who took on an apprentice at Bamburgh for three years in August 1797, for a sum of £105.^49^ Cockayne enjoyed a long tenure at the Castle, and remained in his post for nearly 30 years, despite at times appearing unpopular and, at least once, dishonest.

## Medical Facilities

It seems clear that, with money to spend, John Sharp was keen to equip the Bamburgh facility with the most up-to-date medical technologies. On the one hand, through the trust and indeed his own wealth, Sharp had the financial and executive freedom to select the equipment he wanted without the need to justify it to a board of governors. On the other hand, he had the connections through which to seek advice on the latest medical innovations. The types of equipment at Bamburgh, along with the means through which they were sourced, further emphasise the experimental nature of the charity. Location, and the remoteness of Bamburgh from other medical institutions was an important factor. Also important, however, was a desire to explore and assimilate new medical developments from London.

A key figure in equipping Bamburgh was John Sharp’s brother William, the resident surgeon at St Bartholomew’s hospital and fellow of the Royal Society. In October 1772, William gifted a sedan chair to the infirmary ‘for the use of the hot bath and also for carrying such patients as are weak’.^50^ In June the following year he sent a carriage for the transport of infirm patients.^51^ These were expensive items and demonstrated the early commitment and support that was apparent for Bamburgh. Letters between the two brothers strongly suggest that William Sharp played a key advisory role in matters from equipment purchase to the recruitment of staff. In July 1774, he wrote enclosing ‘a case of vegetable bottles which may be of very great benefit to many in your infirmary’, as well as detailing a new type of tobacco fumigatory.^52^

Also, John Sharp’s youngest brother, Granville, was a regular correspondent of John Coakley Lettsom, the surgeon and philanthropist who had established the first dispensary in London. Granville and Lettsom corresponded on many occasions on matters varying from slavery to philosophy, and it seems likely that Lettsom was consulted about the Bamburgh project.^53^

Once established, the infirmary quickly made links with local suppliers for drugs and equipment. In its early years regular disbursements were made to the Newcastle apothecary John Doughty for large amounts of various medical ingredients, preparations and also surgical instruments.^54^ These were usually sent by chaise to Berwick or Belford where an assistant from the castle collected them. As with the surgeon’s vacancy, it seems that news of a potentially lucrative contract to supply the institution with drugs had reached London. An undated letter from David Taylor, a druggist and chemist of Little Britain, centre of the apothecary business in London, referred to a dispute with Doughty, offering his services as an alternative supplier whence he would ‘make a point of giving satisfaction’.^55^ Some drugs were also bought from Taylor and Seymour in London while, in the 1780s, a regular account was set up with the Newcastle business of ‘Messrs Anderson and Keenlyside’, who supplied everything from lancets to bag trusses for the dispensary.^56^

Several items in the list of fitting-up expenses, however, point to a desire to equip the infirmary with more than simply basic or standard equipment. One early purchase was an ‘electrical machine’, costing over £4.^57^ Medical use of electricity was the ‘coming thing’ in the late eighteenth century. The invention of static electrical generating machines accompanied an increasing belief amongst some prominent physicians, including Erasmus Darwin, that electricity was an elemental substance that could be beneficial to health and healing. Electrifying—electrocuting—patients was thought useful in obstructions and nervous conditions in particular and was used by a range of medical practitioners.^58^ An entry in the accounts for September 1773 noted its repair from ‘being heated with violent use’ and ripped from its mountings!^59^ Two sets of the apparatus for the ‘Recovery of Drowned or suffocated persons’ were another recent invention.^60^ These were a type of bellows that sought to revivify recently deceased bodies using a process of artificial respiration. Given the potential for shipwrecks to occur on the very rocks on which Bamburgh Castle stood, this was a pragmatic purchase. It was also an emerging technology, again suggesting the willingness to try the latest inventions. The ‘infirmary chair used in reducing dislocated shoulders and other operations’ was likely a specialist orthopaedic or postural device of the type becoming more common in the second half of the eighteenth century.^61^ The infirmary also boasted a number of different bathing facilities, including a tumbling seawater bath ‘upon which either spring or seawater is received upon the head’, a cold bath using water pumped from a deep well and a ‘Hot bath with thermometer &c’, which was available to patients and local people for a small fee.^62^

In line with the general model of dispensary expenditure, the amounts spent by Bamburgh trustees were not large. Dispensaries often occupied existing buildings, keeping costs low. The expenses for setting up the Bamburgh infirmary certainly do not suggest a large-scale facility. In the first year of operation, the trustees expended £134.^63^ Although dispensary expenditure was lower than that of hospitals, the Bamburgh figure is in fact less than the usual sum for dispensaries. In 1792 the Public Dispensary in London expenditure totalled £379, whilst at Carlisle in 1800 the figure was £345. Closest to Bamburgh, both in terms of expenditure and proximity, was Whitehaven, which spent £157 in 1800.^64^ Without the costs of actually erecting a purpose-built structure from scratch, costs at Bamburgh could be kept to a minimum. Payments in the first year clearly related to the costs of foundation. This included material alterations to castle rooms to accommodate the surgery equipments. Local craftsmen were paid for a variety of jobs including surgery presses, mounts for pestles and mortars, setting up the infirmary chairs and installing bunting and flags.^65^ The other main expenditure related to equipment, instruments and drugs. Throughout the winter months of 1772/3, large amounts of apothecary drugs were ordered from suppliers in Newcastle and Apothecaries Hall in London. Medicines and substances totalling seven pounds were ordered on one day in December 1772 alone.^66^ An order of drugs from Apothecaries’ Hall on 3 December 1772 amounted to £18, while a variety of other goods from scales to medical books were also bought in.^67^

## The Demographics of Treatment

The admissions register provides useful information about the first 30 or so years of the Bamburgh infirmary.^68^ The register, included numbers of out- and in-patients, patients discharged and relieved, inoculated, deaths, those remaining on the books from the previous year, and an annual total. The exact date of the infirmary’s opening is unclear, but it was almost certainly in operation by the late summer of 1772. In the first year, the infirmary treated 368 out-patients and ten in-patients, of whom 206 were discharged, 68 relieved and ten dead. Even these apparently low numbers appear to have taken the trustees by surprise. Just three months after opening it had become clear that three hours’ attendance by the surgeon was insufficient to deal with patients attending, and this was swiftly increased. A note dated 29 October 1772 suggested that the surgeon’s business could not be carried out in a morning ‘as first expected’, and that his pay was to be increased to allow him to attend full days on Saturdays, and ‘sometimes a part of the preceding and following day’.^69^ The popularity of the infirmary also impressed the trustees. In October 1773, at the end of the first year, the Reverend John Rotherham wrote to John Sharp to express his ‘great pleasure to see the good effects of our charitable institution’, and suggested that a celebratory article be lodged with the *Newcastle Courant*—advice that Sharp did not appear to heed.^70^

**Fig. 1:**
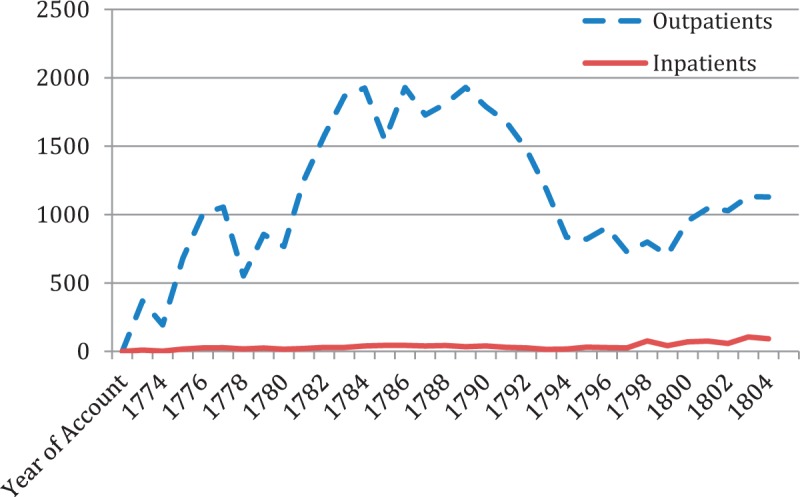
Bamburgh Admissions, 1772–1804

For reasons that are unclear, the numbers of patients fell in the second year to 280. Thereafter, however, the general trend was increasing. Figure 1 shows the numbers of out-patients, in-patients and yearly totals between 1772 and 1804.^71^

In 1775 the total numbers of patients rose by almost two-thirds to 681, and by 1776 they had reached 1009. After dropping back in 1778 they rose dramatically and thereafter remained above 1,000 per year for much of the next decade. In the 1780s the hospital experienced a sustained period of high demand, several times achieving total numbers exceeding 2,000, peaking at 2,073 in 1786. Even if the figures for the number of patients remaining on the books are excluded (i.e. if they are regarded as not new or ‘unique’ patients), the numbers and general pattern are still broadly similar. There are some suggestions that the 1780s witnessed several epidemic outbreaks in the north east, which could partially explain the increasing numbers. The first report of the Newcastle Dispensary contains several references to epidemics, including an outbreak of influenza.^72^ Other evidence demonstrates that this outbreak was widespread, affecting Bamburgh and its locality. In 1782, Captain Read of the nearby Berwick garrison begged John Sharp to allow unaffected soldiers to be quartered at the castle to prevent further infection of his men. Read noted that many of his men were daily falling sick of Influenza, and feared the outbreak would become general.^73^ The Newcastle accounts also refer to ‘epidemic dysentery’ in 1783 and 1785, accounting for hundreds of cases there, and again corresponding to high demand at Bamburgh.^74^ Such outbreaks served to inflate admissions in rural areas as well as densely populated towns.

A second factor behind patterns of admission for which evidence can be found is that of pressures caused by prices. In 1783 George Hall reported ‘the great numbers of people who have resorted to Bambourgh (sic) castle this winter for corn, is a proof of its dearness and scarcity’. Such was the apparent pressure on paupers for food that Hall noted that they often began queuing at the castle gate by three in the morning.^75^ Mobs forming in Berwick the same year caused soldiers from nearby Belford to march there to quell possible food riots. It is also entirely possible that people who came to the castle for corn also took the opportunity to consult the medical personnel. Factors such as hard winters, high food prices and sickness all served to increase pressure on the poor and might account for some of the ‘spikes’ in demand.

Other sources support both the increasing numbers of patients and the added pressures on the dispensary. Initially the trustees viewed the popularity of the institution positively. At the end of its first year of operation, John Rotherham wrote to Sharp extolling the ‘good effects of our Charitable Institution’ and suggested that it ‘ought to be publicised in the Newcastle newspaper at least’.^76^ George Hall’s letters continually remark upon the steady demand for the infirmary’s services. In February 1779 he noted that ‘we had above 50 patients last Saturday’ a number that tested the abilities of Dr Cockayne and his assistants.^77^ By 1782, the strain was beginning to tell. In a letter to John Sharp the doctor referred to the pressures caused by the ‘vast number of patients admitted this year’, and stressed the ‘trouble so great an increase of business must necessarily give me’.^78^ Although the tone was obeisant, and the doctor was keen to avoid writing himself out of a job, he requested a salary rise. The request paid off. Within a year he was granted an annual salary of eighty pounds, in fact at the generous end of salaries for dispensary personnel, which could vary from £30 to £100 in London.^79^

It is also interesting to note the dramatic fall in numbers which began after 1789, and apparently steepened after 1792—the date of Sharp’s death. Although the sources are quiet, it might be speculated that a period of instability caused by Sharp’s death, together with the effects of new governance, affected the intake of the infirmary.

The demand for services raises questions about whether Bamburgh was in any way exceptional in the numbers of patients it treated and, in turn, how its location affected the patient demographic. Providing like-for-like comparisons of admissions with other institutions is problematic. Admission and attendance were greatly affected by local conditions and variables including admissions policies, population size and density, types of condition treated and so on. Nevertheless, some simple comparison is still instructive. The York dispensary, for example, established in 1788, treated around 900 people per year. York was a prosperous market centre of around 17,000 people, with a broad base of wealthy elites and medical practitioners who supported the institution.^80^ The report of the Whitehaven dispensary for the year between June 1783 and 1784 (its opening year) lists 1,467 as ‘recommended and registered’ and a further 433 ‘trivial cases’ giving a round total of 1900.^81^ Whitehaven was the premier port of the Cumberland coalfield with a population of several thousands, described by Charles Creighton as the ‘Newcastle of the west coast’.^82^ Comparing the Bamburgh figures with a neighbouring urban institution over a longer period is also revealing. Between October 1777 and September 1790, 10,866 people visited the Newcastle Dispensary. Using the same criteria as the Newcastle report, between March 1777 and 1790, 22,213 patients visited Bamburgh.^83^ In fact, comparing Bamburgh with the admissions data provided by Irvine Loudon’s survey of dispensaries, confirms that, remarkably, the Bamburgh Castle facility treated as many people in the 1780s as did the General dispensary, Westminster General, and Public Dispensary in London, and the Bristol and Newcastle dispensaries in the provinces.^84^ Comparisons with other dispensaries are more difficult to make on a like-for-like basis and caution must be exercised due to the vagaries of recording admissions. Some do not include extra accident cases or are skewed by variations in admissions policies. The examples listed here, however, are not. Repeatedly, then, in terms of numbers treated, Bamburgh compares with large–even metropolitan–institutions.

Between October 1777 and October 1778, there were a total of 670 full entries in the admissions register.^85^ The published admission figures for institutions are sometimes misleading as evidence of patient numbers, since they actually record sickness episodes. Put simply, if an individual visited an infirmary more than once in any year, their admission would naturally be counted separately each time. As such, the numbers of admissions are not indicators of the actual number of people who sought treatment in any given year.^86^ The effect of this artificial inflation in patient numbers has not been addressed. The Bamburgh admissions register reveals that, out of 670 admissions, 68 people (10 per cent) visited the institution more than once, accounting for 151 (22.5 per cent) admissions. The actual number of individuals using the dispensary in that year was in fact 587. Returning to the point made earlier about parish populations, this discrepancy does go some way towards reconciling the totals, if roughly a fifth of admissions were not from unique visitors. It is also clear that the numbers of visitors fluctuated, sometimes dramatically, although the number of parishioners visiting Bamburgh in the 1780s still appears remarkably high.

How such apparently high patient numbers were sustained therefore bears scrutiny. The parish of Bamburgh covered an area of 48 square miles and contained 22 townships. According to a Victorian survey of Northumberland, the parish population in 1801 was 2,935, with Bamburgh village having 295 inhabitants in the same year.^87^ Given that Bamburgh was reporting admission figures of over 2,000 per year in the 1780s, it might seem unlikely that such an apparently substantial percentage of the parish made use of it. Nevertheless, the relatively small catchment area of the infirmary is established by a list of Bamburgh patients from October 1777 to February 1779 giving names, ages, home parish as well as the condition being treated. This is the only period to contain such detailed information about individuals so it must be regarded cautiously.^88^ Nonetheless, of the 936 names listed, 789 (84 per cent) were from Bamburgh parish, with a further 12 identified as being from the castle—itself regarded as a township. Twenty-two patients each came from the townships of Beadnell and Belford, either within, or against, the parish of Bamburgh. In total, only ten patients (1 per cent) came from outside Bamburgh or its immediate neighbouring parishes. One possibility is that of a relationship between the Bamburgh dispensary and local structures of poor relief. If churchwardens and overseers were officially referring patients to Bamburgh then this might explain the apparent constancy of demand. Given the increasing institutionalisation of poor relief in the latter eighteenth century, this might seem logical. There is, however, no evidence to support such a relationship. Neither the Bamburgh parish chest records nor the churchwardens’ accounts for the last quarter of the eighteenth century contain anything to suggest that any formal relationship was in place.^89^ Poor relief in eighteenth-century north east England has been argued to be patchy in quality and amount, and relatively slow to develop.^90^ There is also anecdotal evidence to support a fairly high proportion of paupers in Bamburgh parish in the last quarter of the seventeenth century but, again, insufficient data exist to corroborate with the later period, making it difficult to assess whether an unusually high number of paupers in the area was the motivation for locating the dispensary in Bamburgh.^91^

The evidence, then, appears to support the relatively narrow extent of Bamburgh’s catchment area. It is certainly possible that the major growth in numbers in the 1780s could have resulted from an extension in its catchment area, as word of its facilities enticed the poor of neighbouring parishes. Equally, however, its relative remoteness may have defined its demographic. For outlying parishes it may simply have been easier and more practical to travel to Newcastle. Indeed, in broad terms, the localised catchment area of Bamburgh actually fits well with the usual model of urban institutions, which were predominantly intended for the use of those in the immediate vicinity. It also, however, suggests that population density was not necessarily a crucial determinant in the popularity of hospitals or dispensaries. In a way, Bamburgh was a hinterland without a town. Without an urban core, the dispensary catered for a scattered, rather than concentrated, population that still numbered in the thousands. Recent studies have laid emphasis upon the mobility of early-modern medical practitioners, but people were also willing to travel to seek medical goods and services, whether through preference (i.e. to see a particular practitioner) or necessity.^92^ The presence, on their doorstep, of a free, well-equipped dispensary was likely a strong inducement for the local population, together with varying numbers of sick poor according to demographic pressures including food prices and disease outbreaks.

## Admission and Treatment

A comprehensive set of records for the first 20 years allow detailed analysis of the progress of the Bamburgh institution, and highlight some interesting variations in terms of admissions, eligibility, treatment and demographics. Currently the subscriber model dominates discussions of admission to eighteenth-century hospitals and infirmaries. Most models relating to the motivations for establishing hospitals and dispensaries are essentially set up to explain subscription systems. As a private charity, with a less restrictive admissions policy, Bamburgh offers a different framework.

As noted above, admission to an eighteenth-century hospital or dispensary required a recommendatory letter signed by one of the institution’s subscribers—a process both practical and symbolic. In practical terms it regulated numbers and prevented the afflicted hoards from descending on the hospital en masse. On another level, however, the need to obtain permission for treatment was a useful means of articulating, asserting and reinforcing social order. In recommending patients, subscribers could adopt a superior position to the ‘worthy objects’ before them who, in turn, were expected to show gratitude for the munificence of their superiors.^93^

At Bamburgh, by contrast, prospective patients required a certificate signed by the minister and two churchwardens of their native parish.^94^ To facilitate this process, the trustees ordered a quantity of printed certificates, leaving blank spaces for the clergy to simply add names and signatures. The document stated that the bearer was a ‘proper object of the charity instituted at Bambrough Castle’ and was ‘not in a capacity to pay for his/her treatment elsewhere’.^95^ Perhaps in line with Sharp’s liberal views on religion, provision was also made for dissenting parishioners, who required the signature of a minister and two elders of their meetinghouse. Although the evidence does not exist to make a detailed comparison of recommendations, it is clear that this was not a straightforwardly Anglican organisation. Exception from a certificate was only made if a patient was recommended directly by one of the trustees, or in emergency cases.^96^ It could be argued that, since admission was still contingent on production of a certificate, Bamburgh was little different to other institutions. Recommendation was ultimately still required and the trustees could act in the same manner as subscribers, perhaps fitting Bamburgh within a similar framework of social relations to that of urban voluntary hospitals and dispensaries.

And yet it was different. On a practical level there was a need to regulate numbers, putting pressure on an already strained staff, so adding the extra layer of difficulty to gaining treatment made sense. Also, however, this was a different relationship, one based on membership of a religious community, rather than a close personal relationship of patronage. Even though, in large rural parishes, seeking a certificate from a local minister may have involved a degree of logistical difficulty, it was arguably, less daunting for a poor parishioner than seeking out a local dignitary, not least because of the personal relationship that already existed. Assuming that the urban poor necessarily had little contact with hospital subscribers is obviously problematic, but the familiarity of the local minister conceivably oiled the wheels of admission. Since the clergy presumably had no financial interest in the Bamburgh dispensary, the usual power dynamics of recommendation also do not appear to fit well. The relative ease by which they could be admitted suggests that Bamburgh patients were neither subject to a patriarchal assertion of status by the trustees, nor were necessarily required or expected to display gratitude to ‘superiors’ or penitence in church. There are no records suggesting that Bamburgh patients were subject to any attempts at moral or spiritual reform that might hint at formal links with parish or institutional poor relief.^97^ Admission for treatment, then, appeared remarkably devoid of caveat, reflecting both the views of its patron and the experimental nature of the castle charity.

There are also differences in the types of treatments offered. Whilst the admissions to voluntary hospitals were mainly surgical cases, dispensaries treated mainly medical conditions. As Irvine Loudon argued, the more exclusive nature of admission in hospitals, together with the lists of conditions they refused to treat, made dispensaries more accessible.^98^ In many cases dispensaries were vocal in their willingness to accommodate patients whose conditions rendered them unsuitable for admission into a voluntary hospital.^99^ Even so, many dispensaries still refused to treat certain conditions, especially contagious fevers and infectious disease. York dispensary, for example, listed smallpox, measles, whooping cough and malignant fever amongst its conditions deemed inadmissible.^100^ Bamburgh was similar in undertaking to treat any and all patients except ‘such as are in a Putrid fever, or such other disorders as may be dangerously infectious’.^101^ Since no specific mention is made of venereal disease, such as the French Pox, it can be reasonably assumed that such conditions were routinely treated.

The vast majority of patients at Bamburgh were outpatients. Whilst there were as many as 19 beds at Bamburgh, it appears that they were for the use of shipwrecked sailors, rather than the sick poor, although there was likely overlap.^102^ In line with other institutions, however, treatment in the patient’s own home was also available, including midwifery services. In 1795, 32 women were ‘attended and delivered at their own homes’ at the expense of the trustees.^103^ Over the following four years, over 100 poor women received midwifery services from the castle surgeon, Mr Herriott.^104^ The Bamburgh trustees were subscribers to the Newcastle Infirmary, giving them the power to refer in-patients to Newcastle if treatment at Bamburgh proved impossible, although this seldom represented more than a few cases per year.^105^ Bamburgh facilities were also held in high enough regard for this arrangement to be reciprocal. In June 1782 the surgeon from the Newcastle Infirmary wrote for permission to send a patient with phthisis and ‘an enormous tumour’ to Bamburgh for treatment because of the quality of its bathing facilities.^106^ In general, though, Bamburgh appears to have been fairly autonomous from other institutions. Whilst many dispensaries were deliberately located to act as auxiliaries to existing hospitals or infirmaries, Bamburgh was created precisely because of the lack of such facilities nearby.^107^

As with many other late eighteenth-century medical institutions, Sharp advocated a programme of inoculation against smallpox. According to the dispensary rules, ‘All children and young persons are inoculated at the Castle & furnished with medicine, who are brought or come there on a surgery day for that purpose.’^108^ This was in place by 1777 and sometimes carried out at the child’s home at the cost of a small gratuity for the doctor. One Mr Turnbull, likely the physician William Turnbull of neighbouring Belford, was paid the sum of three pounds and three shillings for six journeys to inoculate children.^109^ In times of smallpox outbreaks people occasionally wrote to Sharp to ask whether the trustees would be prepared to cover the cost of inoculation.^110^ Here the Bamburgh charity was relatively progressive, adopting the practice largely before the main campaign for the eradication of smallpox in the 1790s. Although the practice had been carried out in Britain since the 1720s, and was beginning to gain acceptance by the 1750s, inoculation in institutions outside London was slow to develop.^111^

What sorts of medical conditions did the Bamburgh dispensary treat? Returning to the patients’ list for 1777/8, the information recorded included the names of admitted patients, their home parish, age at admission, condition, length of treatment and whether cured, relieved or discharged. This record contains a list of over 90 separate conditions, from coughs and colds, to more precise terms such as ‘Anasarca’ [swelling or edema], ‘Erysipelas’ [an acute skin swelling] and ‘Hemiplegia’—today referred to as Cerebral Palsy. Loudon compared the range of conditions treated in the Bristol Infirmary and Bristol Dispensary in 1800. Grouping cases into general categories he found that 98 per cent of dispensary cases were medical, compared with 79.6 per cent in the Infirmary, the rest being surgical.^112^ Whilst the 1800 figures are not available for Bamburgh, it is possible to draw cautious comparisons by comparing the figures for the admissions year beginning October 1777 and using the same general categories of condition used by Loudon (Table 1).

**Table 1.** Comparison of number of admissions Bristol Dispensary, 1800, and Bamburgh Castle, 1778

Type of case	Bristol 1800	Bamburgh 1778
Typhus/putrid fever	367 (38.5%)	13 (1.9%)
Dropsy	33 (3.5%)	4 (0.6%)
Surgical Cases	14 (1.5%)	7 (1.5%)
Genito-urinary disorders	188 (19.7%)	52 (7.8%)
Inflammation and Contusion	8 (0.8%)	16 (2.4%)
Psychological/Mental disorders	15 (1.6%)	29 (4.3%)
Other infective disorders	71 (7.5%)	35 (5.2%)
Sprains/strains/swellings/acute pain	N/A	52 (7.8%)
Diseases of the locomotor system	24 (2.5%)	56 (8.4%)
Skin diseases	0	62 (9.3%)
Other Medical Disorders	51 (5.4%)	74 (11.1%)
Respiratory	158 (16.6%)	99 (14.8%)
Gastro-intestinal	188 (19.7%)	169 (25.3%)
Unknown/undecipherable	N/A	2 (0.1%)
Total	953	670^113^

Note: This figure is for the 12–month period beginning October 1777, rather than the full list noted above, in order to give a like-for-like comparison over a single year.

*Data sources*: Loudon, ‘Origins and Growth’, 337 and NRO 00452/D/5/2/1.

A number of issues are raised. First, as in Bristol, it is clear that medical cases formed the overwhelming majority of admissions at Bamburgh. In Bristol, a combination of surgical cases and inflammation/contusion formed 2.3 per cent of the total. In Bamburgh the figure was only slightly higher at 3.9 per cent. Perhaps more striking, however, is the apparent disparity in treating fevers. In Bristol Dispensary, nearly 40 per cent of treatments related to fevers; at Bamburgh, the number of specific references to fever is just 1.9 per cent. Fevers have been argued to be the mainstay of dispensary admissions.^113^ In crowded urban environments the close proximity of inhabitants, and the often insanitary living conditions, were fertile ground for many epidemic fevers including typhus, typhoid and cholera. It is possible that the rural location and dispersed population of Bamburgh parish rendered its inhabitants less susceptible to this kind of infection. Bamburgh had higher percentages of skin diseases and diseases of the locomotor system than Bristol. It is possible, though difficult to prove, that this category might have included venereal disease. The highest number and percentage of Bamburgh cases related to gastro-intestinal disorders, including vomiting, diarrhoea as well as other stomach complaints including biliousness. The reasons behind a prevalence of these types of conditions at Bamburgh are unclear. One possibility, as was sometimes the case in rural areas, is the generally poor condition of rural housing and living conditions, as well as exposure to disease vectors.^114^ There is no apparent seasonal pattern in that year to suggest an outbreak, implying that other factors, such as diet, played a part. Clearly there are problems in drawing comparisons between different institutions and different time periods, not least of which is the essentially arbitrary nature of the disease groupings. Nonetheless, these figures are at least suggestive of the importance of local conditions in determining the types of treatments offered at different institutions.^115^

Equally, little attention has been paid to the age or gender demographics of those seeking hospital treatment. Here, again, Bamburgh provides some useful evidence. Table 2 shows a breakdown of admissions by approximate life stage, and by gender. Of the 670 admissions, females were only slightly more frequent visitors to the dispensary than males, accounting for 365 (54.5 per cent) episodes. Given its coastal location, and stated aim to care for sick sailors, a trend towards greater numbers of men and boys might have been expected. Nevertheless, the relative parity between all categories of analysis is noteworthy, suggesting that there was no discernibly prominent age or gender group who were more likely to seek treatment. In statistical terms the most numerous visitors, consistent across both genders were between 26 and 40. This might be considered as a life stage of marriage, house holding, childbearing and relative stability. Women of this age display a slightly larger propensity than men to appear as admissions. The age group of 7–14, representing early adolescence and, in some cases, entry to work, make up the second largest group in boys, but is less apparent in girls, for whom the ages of 15–25 are numerically higher. Whilst corresponding with sexual maturity and the onset of menstruation, there is no pattern of illness in this age group, other than a few cases of chlorosis, to suggest this was especially problematic. Children, and especially infants, do not represent a significant proportion of admissions, being roughly concomitant with older and elderly parishioners. Recent work suggests that the numbers of children treated in medical institutions indeed varied greatly according to its size and location. In Bristol Royal Infirmary in 1756 7.7 per cent of patients treated were 13 or under, with 1.5 per cent under 7 years. In Manchester, however, nearly a quarter of patients were under 13, with 15 per cent younger than 7 years.^116^ As elsewhere, children were thought suitable for treatment at Bamburgh, and represented a ‘sizeable minority’.^117^ Overall, the evidence from Bamburgh suggests that the patient demographic was distributed fairly evenly amongst both genders, and across different ages.

**Table 2.** Gender and age range of Bamburgh patients, October 1777–October 1778

Age range Female	Frequency	%
<3	28	8
3–6	29	8
7–14	59	16
15–25	63	17
26–40	85	23
41–50	52	14
51–60	33	9
61–70	10	3
>71	6	2
	365	100

Age range Male	Frequency	%

<3	24	8
3–6	38	12
7–14	54	18
15–25	35	11
26–40	44	14
41–50	33	11
51–60	30	10
61–70	30	10
>71	17	6
	305	100

Age range Total	Frequency	%

<3	52	8
3–6	67	10
7–14	113	17
15–25	98	15
26–40	129	19
41–50	85	13
51–60	63	9
61–70	40	6
>71	23	3
	670	100

*Data source*: NRO 00452/D/5/2/1.

## The Decline of the Bamburgh Castle Dispensary

Before concluding it is useful to briefly consider the longer-term fate of Bamburgh Dispensary. John Sharp died in 1792, having lived long enough to see the great success of his charity. Upon his death it appears that the administration of the castle continued in much the same way, with responsibility passing to Andrew Boult, curate of Bamburgh. A house was built at the castle for Bamburgh’s incumbent, and all of Sharp’s charities were maintained using income from the various estates owned by the Crewe trust, although it is noticeable that the consistency of record keeping diminished rapidly after Sharp’s death.^118^ The immediate decline in the 1790s is not easily explainable from the sources. It is possible that the trustees simply diverted money to other priorities. In the mid 1790s, for example, the nationwide crisis of food supply and prices may have led to the diversion of resources into the provision of cheap corn. A more detailed analysis of the Crewe charity records in the early nineteenth century is needed, although beyond the scope of this article. By the 1860s, however, government inspections of the charity concluded that mismanagement of the trust was leading to a culture of mendicancy, rather than the relief of distress, lamenting the downturn in the fortunes of the institution that John Sharp had founded.^119^ A reduction of income caused by agricultural depression in the final years of the nineteenth century further damaged the capacity of the Bamburgh charities, which were gradually relocated into the surrounding villages. The final payments for medicines for the sick poor of Northumberland ceased in 1958, bringing to an end almost two centuries of charitable medical provision for the sick in Bamburgh.^120^

## Conclusion

As this article has sought to demonstrate, the provision of institutional medical facilities cannot always be understood through the nexus of urban and civic philanthropy. Whilst, in towns, hospitals and dispensaries were bound up in social, political, economic and religious networks, as well as prevailing, patriarchal attitudes towards the poor, the Bamburgh Castle surgery and dispensary, through the patronage and guidance of John Sharp, offers an alternative. While many hospitals and dispensaries, like their infirmary counterparts, could be impressive urban structures, Bamburgh was small, occupying no more than a few rooms in the castle. Located on the coast, 50 miles from the nearest large town, it treated numbers of patients comparable even with large metropolitan institutions. While subscribers and governors usually oversaw the running of large institutions, Bamburgh’s early administration was reducible to Dr Sharp, the trustees, castle bailiffs, practitioners and a few assistants. Whilst its surroundings were literally medieval, its equipment and practices represented the latest in medical thinking.

How, then, are we to understand Bamburgh? In many ways it is so different that it might suggest a straightforward anomaly, in effect the exception to prove the rule. It is certainly difficult to challenge the domination of the urban in hospital provision on the basis of one example. On the other hand, Bamburgh sheds light on a pioneering experiment to apply elements of metropolitan models of medical provision in a unique setting. Obviously aware of broader medical developments, John Sharp took the opportunity to invest heavily in a medical facility, spurred on by the relative remoteness of the parish and distance from other medical institutions. Taking advantage of the relative autonomy offered by the Crewe funds, as well as the physical space of the castle, Sharp was able to combine several medical functions under one roof. Moreover, the records allow us an insight into the progress of this experiment over a quarter of a century.

Whilst the question of centres and peripheries is implicit within much hospital historiography, in terms of the communities served by them, far less attention has been paid either to the size of hinterlands for town institutions, or to the availability of hospital services to the rural poor. Indeed, so embedded are hospitals within urban frameworks that it is difficult to fully contextualise a medical institution not linked to a town. This was an institution riven with apparent paradoxes, not least of which was its location. But such problems raise questions about the form, function and location of medical institutions. As this article has argued, Bamburgh fits none of the usual compartmentalised models of medical provision. In many respects it fits the cottage hospital model, although predating it by many decades. Why, though, were there no other similar examples of rural institutions during this period, so far as we are aware? Perhaps Sharp’s experiment was born of a unique confluence of circumstances, not easily replicated elsewhere. It also, however, suggests that renewed attention needs to be paid to other types of rural institution, for example workhouses, to establish if parallels can be discerned, or whether the demographic and social conditions in Northumberland were replicated in other parts of Britain at the time, with scattered populations not served by an urban centre.

In this respect, Bamburgh indeed offers new insights into towns and hinterlands, and the provision of medicine to the rural poor. The sheer numbers of admissions, for example, suggest that population density was not a prerequisite for the ‘success’ of an institution. Bamburgh was a rural parish whose inhabitants were prepared to use local facilities, even if that meant travelling a few miles. This, in turn, raises further questions about the tensions between institutional ‘mission statements’ and actual patient demographics. Whilst many institutions paid lip service to the treatment of ‘strangers’ (in Bamburgh’s case shipwrecked sailors), the overwhelming majority of patients lived within a few miles of the institution. More work is needed to address both definitions and treatment of ‘strangers’.

To make value judgements about the quality of medical treatment simply by the availability of facilities is clearly unsafe. A sceptic might view the expensive and ‘modern’ medical technologies available at Bamburgh as little more than conspicuous consumption made possible by the extensive Crewe charity funds. But, as we have seen, a wealth of evidence points to the extensive use of the institution in the last quarter of the eighteenth century. Other factors, from the close relationship with Saint Bartholomew’s hospital via William Sharp, to the apparently high estimation in which the Bamburgh facility was held both by fellow practitioners and other institutions, as well as the apparently low mortality rates noted in the admissions register, all build a picture of a facility that was, paradoxically, both remote and extremely well connected.

## Footnotes

1 Northumberland Archives (hereafter NRO), Lord Crewe Trust Records MS 00452/D/8/2/11/42, records of trustee decisions.

2 NRO 00452/D/8/4/8/4 ‘An account of the uses to which the old Roman Tower in Bambrough Castle is at present appropriated’, Undated *c*. 1772.

3 For general examples see Susan C. Lawrence, *Charitable Knowledge: Hospital Pupils and Practitioners in Eighteenth-Century London* (Cambridge: Cambridge University Press, 1996); John Woodward, *To Do the Sick No Harm: A Study of the British Voluntary Hospital System to 1875* (London: Routledge and Kegan Paul, 1974); John Henderson, Peregrine Horden and Alessandro Pastore, *The Impact of Hospitals, 300–2000* (London: Peter Lang, 2007); Lindsay Granshaw and Roy Porter (eds.), *The Hospital in History* (London and New York: Routledge, 1989); Dana Arnold, *The Spaces of the Hospital: Spatiality and Urban Change in London, 1680–1820* (London: Routledge, 2013).

4 Bronwyn Croxson, ‘The Public and Private Faces of Eighteenth-Century London Dispensary Charity’, *Medical History*, 41 (1997), 131–3.

5 Adrian Wilson, Conflict, Consensus and Charity: Politics and the Provincial Voluntary Hospitals in the Eighteenth Century', *English Historical Review*, 111 (1996), 599.

6 Roy Porter, ‘The Gift Relation: philanthropy and provincial hospitals in eighteenth-century England’ in Lindsay Granshaw and Roy Porter (eds.), *The Hospital in History* (London: Routledge, 1989), 155.

7 Wilson, ‘Conflict’, 602.

8 Patrick Wallis, ‘Charity, Politics and the Establishment of York County Hospital: A “Party Job”?’, *Northern History*, 48 (September 2001), 250–1.

9 Croxson, ‘Public and Private Faces’, 128–9.

10 *Ibid*.

11 Irvine Loudon, ‘The Origins and Growth of the Dispensary Movement in England’, *Bulletin of the History of Medicine*, 55 (1981), 325.

12 Robert Kilpatrick, ‘“Living in the Light”: Dispensaries, Philanthropy and Medical Reform in Late Eighteenth-Century London’, in Andrew Cunningham and Roger French, eds, *The Medical Enlightenment of the Eighteenth Century* (Cambridge: Cambridge University Press, 1990), 257; see also the discussion of dispensary functions in Croxson, ‘Public and Private Faces’, 128–9.

13 Kilpatrick, ‘Living in the Light’, 257–8.

14 For a useful discussion of the Newcastle Dispensary, see Graham A. Butler, ‘Disease, Medicine and the Urban Poor in Newcastle Upon Tyne, c. 1750–1850’ (unpublished PhD thesis, Newcastle University, 2012), 147–95; F. J. W. Miller, ‘The Newcastle Dispensary, 1777–1976’, *Archaeologia Aeliana*, 5 (1995), 177–96.

15 For example Kilpatrick, ‘Living in the Light’; Croxson, ‘Public and Private Faces’.

16 Françoise Deconinck-Brossard, ‘Sharp, John (1723–1792)’, *Oxford Dictionary of National Biography*, Oxford University Press, 2004 <http://0-www.oxforddnb.com.lib.exeter.ac.uk/view/article/65613>, accessed 9 May 2014.

17 C. J. Stranks, *The Charities of Nathaniel, Lord Crewe and Dr John Sharp, 1721–1976* (Durham: Durham Cathedral Lecture, 1976), 14.

18 William Hutchinson, *A View of Northumberland: With an Excursion to the Abbey of Mailross in Scotland* (London: Printed by T. Saint, 1776), 178.

19 NRO MS 9068, Benjamin West (attributed), Portrait of Dr Sharp, undated.

20 Thomas Newcome, ed., *The Life of Dr John Sharp, D.D., Lord Archbishop of York …* (London: C. and J. Rivington, 1825), 270.

21 NRO MS 452/B/1/6, Lord Crewe Charity, Castle notebook, 20 August 1772, p. 141.

22 Woodward, ‘*To do the Sick no Harm*’, 11.

23 Anon/Royal Infirmary of Edinburgh, *An Account of the Rise and Establishment of the Infirmary, or Hospital for Sick Poor, at Edinburgh* (Edinburgh: publisher unknown, 1730), 1–2.

24 Thomas Talbot, *Three Addresses to the Inhabitants of the County of Hereford* (Hereford: C. Pugh, 1774), 4.

25 Donna T. Andrew, *Philanthropy and Police: London Charity in the Eighteenth Century* (Princeton, NJ: Princeton University Press, 1989), 136, 155, 201.

26 *Ibid*., 324.

27 *Ibid.*, 137.

28 *Ibid*., 201.

29 Jonathan Reinarz and Leonard Schwarz, ‘Introduction’, in Jonathan Reinarz and Leonard Schwarz, eds, *Medicine and the Workhouse* (Rochester: University of Rochester Press, 2013), 3–5.

30 Ephraim Chambers, *Cyclopædia: or, an universal dictionary of arts and sciences, Volume 2* (London: Printed for W. Strahan et al, 1778), 424.

31 *Ibid*., 426.

32 *Ibid*., 545.

33 *Ibid*.

34 Anon, *Encyclopædia Britannica; or, a dictionary of arts, sciences, and miscellaneous literature*, 3rd edn (Edinburgh, printed for A. Bell, 1797), 52.

35 Loudon, ‘Origins and Growth’, 333, n. 28.

36 George Alexander Cooke, *Topographical and Statistical Description of the County of Durham* (London: Printed for C. Cooke, date unknown, *c*. 1820), 39 [my emphasis].

37 NRO MS 452/B/1/6, Lord Crewe Charity, Castle notebook, 20 August 1772, p. 141.

38 *Ibid*.

39 NRO, MS 452, Lord Crewe Trustee Papers, Undated—‘Very Important Memoranda in this book respecting Bamburgh Castle ancient and modern’, f. 20.

40 See Irvine Loudon, ‘John Bunnell Davies and the Universal Dispensary for Children’, *BMJ*, 5 (1979), 1191–94.

41 Hutchinson, *View of Northumberland*, 178.

42 Carol Griffiths, *Bamburgh's Ghosts: Voices from the Eighteenth Century* (Bamburgh: Privately printed, 2014), 8.

43 NRO MS 00452 B/1/6, ‘Important Memoranda’, 20 August 1772.

44 *Ibid*.

45 See P. J. Wallis and R. V. Wallis, eds, *Eighteenth Century Medics* (Newcastle upon Tyne: Project for Historical Bibliography, 1988), 608.

46 NRO MS 00452/C/3/2/5/41, Letter from ‘Mr Gair’ to Dr John Sharp, 25 June 1774. For Gairs see Wallis and Wallis, *Eighteenth Century Medics*, 218.

47 NRO MS 00452 C/3/2/5/44, Letter from R’ev’d Mr Potter Wallend’, to Dr John Sharp, 3 June 1744.

48 NRO MS 00452/C/3/2/5/69, Letter William Sharp to John Sharp, 14 September 1774.

49 Wallis and Wallis, *Eighteenth Century Medics*, 122.

50 *Ibid*.

51 NRO MS 00452 D/5/2/1/1/, 1.

52 NRO MS 00452 C/3/2/5/51.

53 Thomas J. Pettigrew, *Memoirs of the Life and Writings of the Late John Coakley Lettsom, with a selection of his correspondence, Volume 2* (London: Printed by Nichols and Son, 1817), 234–48.

54 *Ibid*., 1.

55 NRO MS 00452/C/3/2/14/66, Letter from David Taylor, Druggist & Chemist no. 52 Little Britain, to Dr John Sharp, undated.

56 NRO MS 00452 D/5/2/1, 117.

57 NRO MS 00452 D/5/2/1.

58 Paul Elliott, ‘“More Subtle than the Electric Aura”: Georgian Medical Electricity, the Spirit of Animation and the Development of Erasmus Darwin’s Phsycophysiology’, *Medical History* 52 (2008), 198–200.

59 NRO MS 00452/D/5/2/1/1, p. 25.

60 See for example John Hunter, ‘Proposals for the Recovery of People Apparently Drowned’, *Philosophical Society Transactions*, 1 January 1776, 423–5; Carolyn D. Williams, ‘“The Luxury of Doing Good”: Benevolence, Sensibility and the Royal Humane Society’, in Roy Porter and Marie Mulvey-Roberts, eds, *Pleasure in the Eighteenth Century* (Basingstoke: Macmillan, 1996), 77–107.

61 NA 00452/D/8/4/8/4. For eighteenth-century orthopaedic devices see David M. Turner and Alun Withey, ‘Technologies of the Body: Polite Consumption and the Correction of Deformity in Eighteenth-Century England’, *History*, 99 (2014), 775–96.

62 *Ibid*.

63 It is worth noting that the large and highly detailed register of expenditures would support a full, long-term analysis, for example of the percentages expended on medicines, equipment, care, staff and so on over time, although such a study is beyond the scope of this article.

64 Loudon, ‘Origins and Growth’, 339.

65 NRO MS 00452/D/5/2/1/1, Memoranda of expenses for fitting up the surgery at Bamburgh Castle, n.d.

66 *Ibid.*, 2.

67 *Ibid*.

68 NRO MS NRO 00452/D/8/2/11/4, List of patients at Bamburgh Castle Infirmary, October 1772.

69 *Ibid*.

70 NRO MS 00452/D/8/2/11/6, Letter from John Rotherham to John Sharp, 25 October 1773.

71 Joan Lane mentions Bamburgh in passing, and quotes a figure of 572 outpatients for 1782—a figure greatly at variance with the totals from the admissions register and indeed Bamburgh’s own figures published in the *Newcastle Courant*. No reference for the source of this information is given, so it cannot be verified. See Joan Lane, *A Social History of Medicine: Health, Healing and Disease in England, 1750–1950* (London: Routledge, 2001), 90.

72 Anon, *An Account of the Newcastle Dispensary for the Relief of the Poor, from its Commencement in 1777, to Michelmas 1789* (Newcastle: 1789), 10.

73 NRO MS 00452 C/3/2/12/15, Letter from Captain Read to Dr John Sharp, 14 June 1782.

74 Anon, *Account of the Newcastle Dispensary*, 10.

75 Quoted in Griffiths, *Bamburgh’s “Ghosts”*, 36.

76 NRO MS 00452 D/8/2/11/16, letter from ‘Revd Mr Rotherhan’ to John Sharp, 25 October 1773.

77 NRO MS 00452 C/3/2/8/46, letter from George Hall to John Sharp, 6 February 1779.

78 NA 00452 C.3.2.12.36, letter from William Cockayne to John Sharp, 17 October 1782.

79 Loudon, ‘Origins and Growth’, 329.

80 Katherine A. Webb, ‘“One of the Most Useful Charities in the City” York Dispensary 1788–1988’, University of York, *Borthwick Paper No. 74*, 6–7.

81 Quoted in Michael Sydney, *Bleeding, Blisters and Opium: Joshua Dixon and the Whitehaven Dispensary* (Workington: Stainburn, 2009), 52–3.

82 Charles Creighton, *A History of Epidemics in Britain: From A.D. 664 to the Great Plague, Volume 1* (Cambridge: 1891), 152.

83 F. J. W. Miller, ‘The Newcastle Dispensary 1777–1976’, *Archaeologia Aeliana*, 5th Series, 18 (1995) 181.

84 Loudon, ‘Origins and Growth’, 327.

85 NRO 00452/D/5/2/1, On acct of carrying on the Charitable Institution at Bambrough Castle for the relief of the Sick and Lame poor, August 1772.

86 *Ibid*., 328–9.

87 William Whelan & Co., *History, Topography and Directory of Northumberland comprising a General Survey of the County …* (London: Whittaker and Co., 1855), 560.

88 NRO 00452 D/5/2/1/1, Memoranda of Expenses, undated, 262.

89 NRO MS EP59/52, Churchwardens accounts Bamburgh parish, 1724–1775.

90 Peter Rushton, ‘The Poor Law, The Parish and the Community in North East England, 1600–1800’, *Northern History* 25 (1989), 137.

91 *Ibid*., 143.

92 For example, Ian Mortimer, *The Dying and the Doctors: The Medical Revolution in Seventeenth Century England* (London: Boydell and Brewer, 2009).

93 Croxson, ‘Public and Private Faces’, 134–5.

94 NRO MS 00452/D/8/2/11/1, ‘Rules to be Observed by the Surgeon on the Commencement of Their Additional Salary’, 23 June 1785. As a point of interest, the rules were still in use in 1829, when an incoming surgeon re-signed the document.

95 NRO MS 00452 D/8/2/11/13, Form of a petition for admission of patient to Bamburgh Infirmary, undated, *c*. 1772.

96 NRO MS 00452/D/8/2/11/1.

97 This was certainly a feature of some voluntary hospitals. See, for example, Wallis, ‘Establishment of York County Hospital. 247–8.

98 Loudon, ‘Origins and Growth’, 334–5.

99 For example Edinburgh Dispensary, quoted in Croxson, ‘Public and Private Faces’, 135.

100 Webb, ‘One of the Most Useful Charities’, 2.

101 NRO 00452/D/8/4/8/4.

102 Lane, *Social History*, 90.

103 NRO MS 00452 D/5/2/1/1, 165.

104 *Ibid*., 185, 186, 193.

105 Webb, ‘One of the Most Useful Charities’, 2.

106 NRO MS 00452/C/3/2/12/12, Letter from Dr Rotherham at Newcastle Infirmary to Dr Sharp, 13 June 1782.

107 Loudon, ‘Origins and Growth’, 335.

108 NRO MS 00452/D/8/4/8/4, Undated ‘An account of the uses to which the old Roman Tower in Bambrough Castle is at present appropriated’.

109 NRO MS 00452 D/5/2/1/1, 48; For Turnbull see Wallis and Wallis, *Eighteenth Century Medics*, 606.

110 For example see NRO MS 00542/C/3/2/6/53 Letter from ‘Miss Graham’ to John Sharp, 22 November 1776.

111 Adrian Wilson, ‘The Politics of Medical Improvement in Hanoverian London’, in Cunningham and French, eds, *The Medical Enlightenment of the Eighteenth Century*, 25, 33–4.

112 Loudon, ‘Origins and Growth’, 337.

113 *Ibid*., 332–3.

114 This was certainly a characteristic of early-modern rural disease demography. See Alun Withey, *Physick and the Family: health, medicine and care in Wales, 1600–1750* (Manchester: Manchester University Press, 2012), 17–18.

115 See Erika Dyck and Christopher Fletcher, *Locating Health: Historical and Anthropological Investigations of Place and Health* (London: Pickering and Chatto, 2010).

116 Alysa Levene, Jonathan Reinarz and Andrew Williams, ‘Child Patients, Hospitals and the Home in Eighteenth-Century England’, *Family and Community History*, 15 (2012), 19.

117 *Ibid*., 20.

118 Stranks, *The Charities of Nathaniel, Lord Crewe*, 17.

119 *Ibid*., 20.

120 *Ibid*., 20, 24.

